# The causal effect of air pollution on the risk of essential hypertension: a Mendelian randomization study

**DOI:** 10.3389/fpubh.2024.1247149

**Published:** 2024-02-15

**Authors:** Zhiwei Xia, Yinjiang Liu, Chao Liu, Ziyu Dai, Xisong Liang, Nan Zhang, Wantao Wu, Jie Wen, Hao Zhang

**Affiliations:** ^1^Department of Neurology, Hunan Aerospace Hospital, Changsha, Hunan Province, China; ^2^Department of Neurosurgery, The Second Affiliated Hospital, Chongqing Medical University, Chongqing, China; ^3^Department of Neurosurgery, Central Hospital of Zhuzhou, Zhuzhou, Hunan Province, China; ^4^Department of Neurosurgery, Xiangya Hospital, Central South University, Changsha, Hunan Province, China; ^5^National Clinical Research Center for Geriatric Disorders, Xiangya Hospital, Central South University, Changsha, Hunan Province, China; ^6^College of Life Science and Technology, Huazhong University of Science and Technology, Wuhan, Hubei, China; ^7^Department of Oncology, Xiangya Hospital, Central South University, Changsha, Hunan Province, China

**Keywords:** PM2.5, PM10, hypertension, Mendelian randomization (MR), air pollution

## Abstract

**Background:**

Air pollution poses a major threat to human health by causing various illnesses, such as cardiovascular diseases. While plenty of research indicates a correlation between air pollution and hypertension, a definitive answer has yet to be found.

**Methods:**

Our analyses were performed using the Genome-wide association study (GWAS) of exposure to air pollutants from UKB (PM2.5, PM10, NO_2_, and NO_X_; *n* = 423,796 to 456,380), essential hypertension from FinnGen (42,857 cases and 162,837 controls) and from UKB (54,358 cases and 408,652 controls) as a validated cohort. Univariable and multivariable Mendelian randomization (MR) were conducted to investigate the causal relationship between air pollutants and essential hypertension. Body mass index (BMI), alcohol intake frequency, and the number of cigarettes previously smoked daily were included in multivariable MRs (MVMRs) as potential mediators/confounders.

**Results:**

Our findings suggested that higher levels of both PM2.5 (OR [95%CI] per 1 SD increase in predicted exposure = 1.24 [1.02–1.53], *p* = 3.46E-02 from Finn; OR [95%CI] = 1.04 [1.02–1.06], *p* = 7.58E-05 from UKB) and PM10 (OR [95%CI] = 1.24 [1.02–1.53], *p* = 3.46E-02 from Finn; OR [95%CI] = 1.04 [1.02–1.06], *p* = 7.58E-05 from UKB) were linked to an increased risk for essential hypertension. Even though we used MVMR to adjust for the impacts of smoking and drinking on the relationship between PM2.5 exposure and essential hypertension risks, our findings suggested that although there was a direct positive connection between them, it is not present after adjusting BMI (OR [95%CI] = 1.05 [0.87–1.27], *p* = 6.17E-01). Based on the study, higher exposure to PM2.5 and PM10 increases the chances of developing essential hypertension, and this influence could occur through mediation by BMI.

**Conclusion:**

Exposure to both PM2.5 and PM10 is thought to have a causal relationship with essential hypertension. Those impacted by substantial levels of air pollution require more significant consideration for their cardiovascular health.

## Introduction

As industrialization advances daily since its inception, air pollution has become an alarming environmental issue that severely endangers modern living standards and public health (1, 2). Approximately 7 million fatalities can be attributed solely to air pollution, according to estimates made by the Global Burden of Disease Study (3). The major constituents of air pollution are particles along with various types of gases (4), while the classification for these tiny solid elements is determined by their size <10 microns [PM10] and < 25 microns [PM2.5], (5). The most significant sources of atmospheric pollution from gas components include NO_X_ and NO_2_, which arise from burning fossil fuels at high temperatures (6).

Essential hypertension, a predominant contributor to global morbidity and mortality (7), has witnessed an incremental prevalence in adults, surpassing 30 % post-2010 (8). Effective interventions are urgently needed in low-income countries due to their anticipated heavy burden of hypertension on healthcare systems (9), and essential hypertension is responsible for more than 90% of all reported cases of high blood pressure (10). Recognized established risk factors for hypertension include a diet that is high in sodium but low in potassium (11), lack of physical activity (12), and obesity (13).

The relationship between hypertension and air pollution has been extensively researched over the past decade, with multiple cross-sectional and cohort studies being conducted. Still, the final results are not entirely consistent (14). Various studies have varied in assessing the effects of different air pollutants on hypertension, and some have even been widely divergent (15–17). This may be due to the limitations of most epidemiological evidence, such as the inability to determine the causal relationship between PM2.5 pollution and hypertension, which may lead to confusion in causality; the data used in the studies mostly comes from regions or communities, rather than individuals; and potential confounding factors such as participants’ diet, physical activity, and lifestyle cannot be excluded.

The introduction of genetic polymorphisms opens a new frontier in investigating air pollution’s impact on health. Genetic variations among individuals can significantly modulate the body’s response to pollutants, potentially influencing the onset and progression of conditions like hypertension. This variability underscores the need for a more nuanced understanding of the interplay between genetic factors and environmental exposures (18).

This study carves a novel path by leveraging Mendelian randomization (MR)—a technique that circumvents traditional observational study pitfalls using genetic polymorphisms as instrumental variables to infer causality. MR parallels randomized controlled trials in design, nullifying biases from reverse causality since genotypes are not modifiable by disease states (19). In principle, the MR analyses rely on three basic assumptions: First, the genetic variants should present a robust association with the exposure. Second, the genetic association between the exposure and outcome should be independent of confounders. Third, the genetic variants affect the outcome exclusively via the exposures (20, 21). To deepen our analysis, we employ multivariable Mendelian randomization (MVMR) (22), a method not yet widely applied in this context, to examine the direct effect of air pollution on hypertension after adjusting for common risk factors, including obesity, smoking, and alcohol consumption.

In this study, we performed two-sample MR analyses to investigate the causal relationship between four air pollutants (PM2.5, PM10, NO_X_, NO_2_) and essential hypertension. Although one-sample MR is likely to be biased by the overlapping population between the datasets of exposures and outcomes, recent researches suggest that this potential overlap may not bias the results as previously thought (20, 23). Thus, we performed one-sample MRs to replicate the analysis for two-sample MRs to confirm the validation. To take some confounders and mediators into consideration, we conducted MVMR analyses to explore the more direct causality.

## Methods

### Study design and GWAS summary data

**Figure 1:**
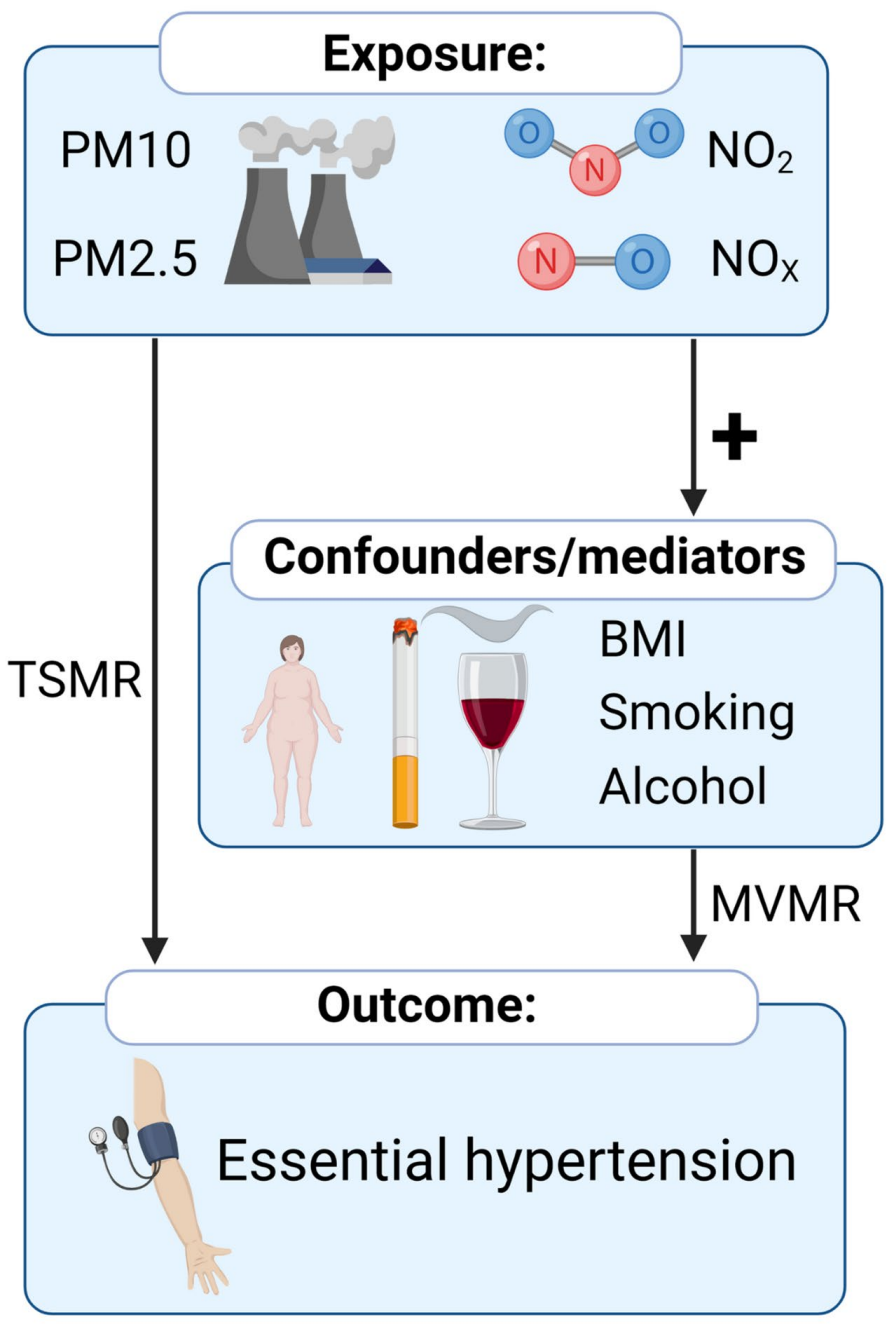
Overview of the Mendelian randomization study of the causal relationship between air pollution and hypertension.

The flow chart of the study design is shown in Figure 1. All summarized Genome-wide association study (GWAS) summary data for each respective phenotype were obtained from the publicly available datasets (MRC IEU OpenGWAS) (24).^1^ The corresponding GWAS ID and basic information about the included GWAS are shown in Supplementary Table 1. The procedure for extracting IVs from summarized GWAS was followed by the “TwoSampleMR” R package^2^ through GWAS ID (24). No restriction of gender, age, income, or education level was set for these GWAS.

The summarized GWAS data of participants living in different air pollution areas were derived from UK Biobank (25–27). The extent of residential air pollution was estimated in different sites in Great London by a land use regression for the annual average 2010. The mean PM10 was 16.24 ± 1.90micro-g/m3, ranging from 11.78 to 31.39 micro-g/m3. The mean PM2.5 was 9.99 ± 1.06 micro-g/m3, ranging from 8.17 to 21.31 micro-g/m3. The summary-level GWAS of PM10 and PM2.5 included 423,796 individuals and 9,851,867 single-nucleotide polymorphisms (SNPs). The mean NO2 was 26.71 ± 7.58 micro-g/m3, ranging from 12.93 to 108.49 micro-g/m3. The mean NOx was 44.11 ± 15.53 micro-g/m3, ranging from 19.74 to 265.94 micro-g/m3. The summary-level GWAS of NO2 and NOx both included 456,380 individuals and a total of 9,851,867 SNPs.

The GWAS data for the potential confounders or mediators, including body mass index (BMI), alcohol intake frequency, and the number of cigarettes previously smoked daily, were also obtained from the UK biobank (24, 25, 28), which included 336,109 participants with 10,894,596 SNPs, 336,965 participants with 10,894,596 SNPs and 78,291 participants with 10,894,596 SNPs, respectively.

For the outcome, the summarized GWAS of essential hypertension in the FinnGen study (release 5) was selected to avoid sample overlapping, generated from longitudinal phenotype and digital health records produced by national health registries (29). This GWAS included 42,857 patients with essential (primary) hypertension, diagnosed according to the International Classification of Diseases (ICD) diagnosis codes (version 10), and 162,837 controls with 16,380,466 SNPs. For the GWAS used for validation, the GWAS of essential hypertension in the UK Biobank (UKB), a prospective cohort recruited from the UK general population between 2006 and 2010, was selected. This GWAS included 54,358 patients with self-reported physician-diagnosed essential (primary) hypertension (PheCode 401.1) and 408,652 controls with 9,851,867 SNPs (24, 25). The large sample size of UKB could further validate the results and enhance the statistical power. As the outcome variable was binary (whether they have hypertension), the effect size of each SNP in the summarized GWAS was represented as beta [log (OR)].

### Selection for instrumental variables

To maintain sufficient instrumental variables (IVs) in MR, we set the value of p threshold for genome-wide correlations as 5e-6 to select solid instrumental variables (IVs) (30). Then, linkage disequilibrium analysis (r^2^ < 0.001, distance <10 MB) based on the 1,000 Genomes Project of the European samples was performed to select independent IVs. IVs strongly associated with the outcome were excluded to meet the MR assumption. F statistics, as an indicator of weak IVs, were calculated by (R2/K)/[(1-R2)(N-K-1)] for each IVs, where K is the number of SNP, N is the sample size, R2 is the variance explained by SNPs calculated by 2*EAF*(1-EAF)*(Beta/SE)^2^ (31). IVs with *F* < 10 were excluded to maintain the robustness. Harmonization of IVs was performed by the function of “harmonise_data” in “TwoSampleMR” R package to ensure that the association estimates of genetic variants aligned with the effect of the same allele between exposure and outcome GWAS (24).

### Univariable Mendelian randomization

We used three methods (random-effects inverse variance weighting (IVW), weighted median, and MR egger) for TSMR analysis, with IVW as the primary approach and the other two as supplements. IVW provided a weighted regression of IVs outcome effects on exposure effects under the assumption of constrained intercept to zero, which owned the optimal statistical power. However, if horizontal pleiotropy existed in IVs, causal pathways other than exposure would interfere with the outcome. Thus, we supplemented the other two methods, which were relatively robust to horizontal pleiotropy, although the statistical power was partially sacrificed (32). The approach of weighted median selected median MR estimates for causal estimation (33). For MR Egger regression, the intercept was allowed to be estimated freely as a measure of average pleiotropy (34).

To estimate the robustness of the results, we performed analyses for horizontal pleiotropy, including leave-one-out tests and MR egger intercept test of deviation from null (35). The tests differed in their underlying presumptions but fundamentally gaged the degree to which the impact of one or more instrument SNP is overblown in magnitude, operating through the hypothesized pathway and other unaccounted-for-for causal pathways.

### Multivariable Mendelian randomization

Multivariable Mendelian randomization (MVMR) allowed for estimating the effects of multiple exposures on an outcome, which depended on the covariance between the effect of the IV on each included exposure (36, 37). In this study, we performed MVMR to investigate the potential mediating role of common risk factors (BMI, alcohol intake frequency, and the number of cigarettes previously smoked daily) in the pathway from air pollution to hypertension. As the number of variables included in MVMR increases, the power of the MVMR would decrease (37). Thus, our MVMR model only included one type of air pollution and one additional risk factor for each analysis. The extraction of IVs, clump process, and harmonization followed the same procedure as univariable MR, as described before. IVs significantly associated with the outcome were excluded.

The MVMR estimates the direct causal effect of the exposure on the outcome adjusting for the mediator, while the univariable MR estimates the total causal effect. The difference between the total causal effect of air pollution on hypertension (from univariable MR) and the direct causal effect (from MVMR) would indicate a mediating role of the common factor. The indirect effect was not calculated because the linear relation between the exposure and outcome, which is required for the estimation of indirect effect, was not secured due to the binary variable of the outcome (38, 39).

All the statistical analyses were conducted in R software (40) by R package “TwoSampleMR” (24) and visualized by R package “ggplot2” (41).

## Results

### Genetic instruments

After a series of filter processes, we extracted 64, 34, 96, and 83 IVs proxing PM2.5, PM10, NO2, and NOx, respectively (Supplementary Tables 2–5). All the F statistics of the IVs were above 10, suggesting the absence of weak instrument bias.

### Univariable MR analysis and sensitivity analyses

**Figure 2:**
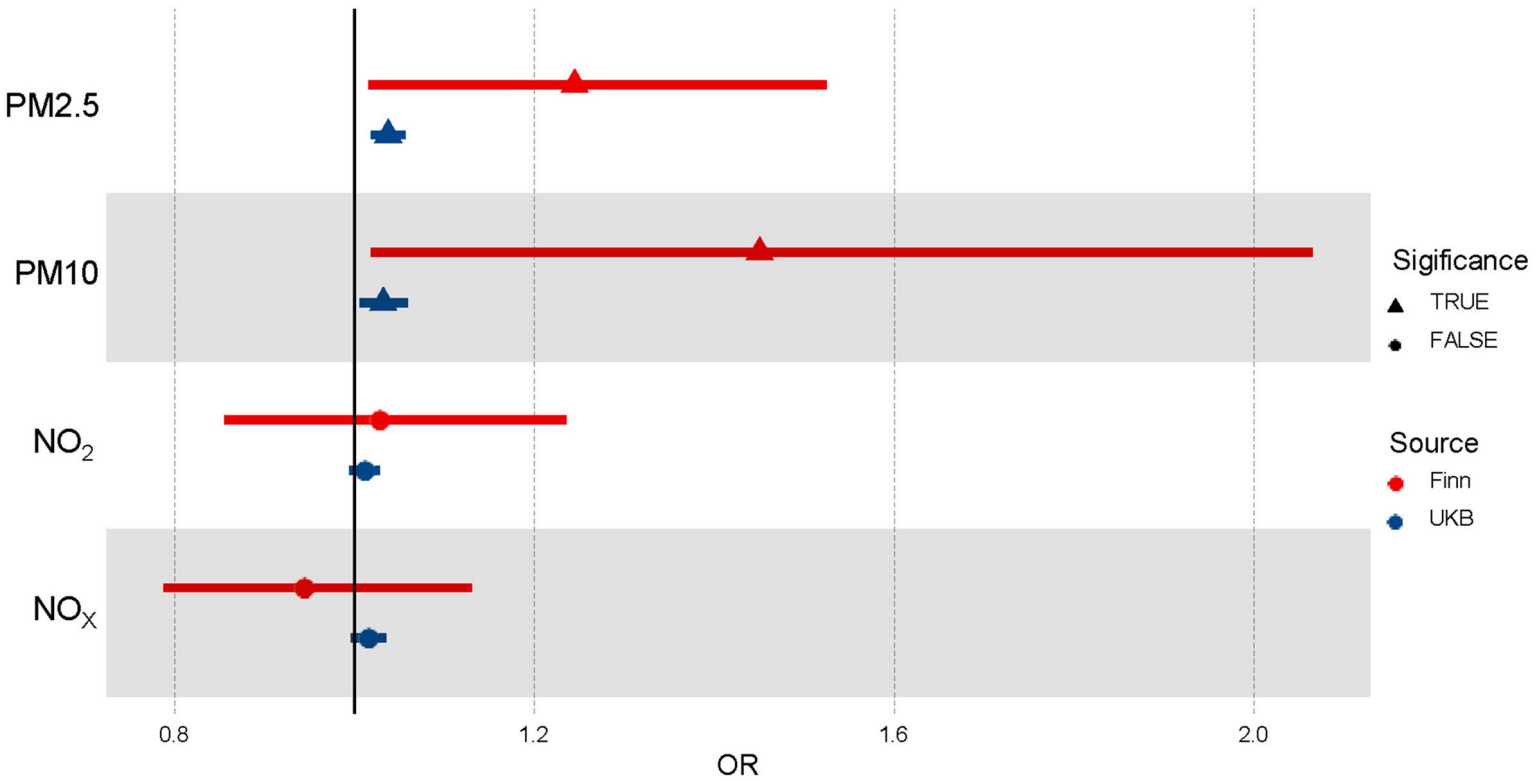
Forest plot of the association between air pollutants and hypertension using Univariable Mendelian randomization. OR means odds ratio. TRUE represents the causal association between the air pollutant and hypertension. FALSE represents no significant causal association between the air pollutant and hypertension.

We conducted univariable MR analyses for PM2.5, PM10, NO_2_, and NO_X_ on essential hypertension separately by inverse variance weighted, MR Egger, and Weighted median to investigate the causal effects of air pollution on essential hypertension (Supplementary Table 6). Results (Table 1; Figure 2) by IVW showed that there was a positive correlation between the increase of PM2.5 and the occurrence of essential hypertension (OR [95%CI] per 1 SD increase in predicted exposure = 1.24 [1.02–1.53], *p* = 3.46E-02 from Finn; OR [95%CI] = 1.04 [1.02–1.06], *p* = 7.58E-05 from UKB). The effect of PM10 on essential hypertension was also significant (OR [95%CI] = 1.45, [1.02–2.07], *p* = 3.92E-02 from Finn; OR [95%CI] = 1.03 [1.01–1.06], *p* = 1.70E-02 from UKB). However, the effects between NO_2_ (OR [95%CI] = 1.03 [0.86–1.24], *p* = 7.70E-01 from Finn; OR [95%CI] = 1.01 [0.99–1.03], *p* = 5.30E-01 from UKB), NO_X_ (OR [95%CI] = 0.94 [0.79–1.13], *p* = 5.30E-01 from Finn, OR [95%CI] = 1.02 [1.00–1.03], *p* = 1.17E-01 from UKB) and essential hypertension were weak or nonexistent.

**Table 1:** MR results for causal effects of air pollution on essential hypertension by IVW.

Exposure	Outcome	Source	nSNP	OR	LCI	UCI	pval
PM2.5	Essential hypertension	Finn	59	1.24	1.02	1.53	3.46E-02
		UKB	61	1.04	1.02	1.06	7.58E-05
PM10		Finn	33	1.45	1.02	2.07	3.92E-02
		UKB	33	1.03	1.01	1.06	1.70E-02
NO_2_		Finn	90	1.03	0.86	1.24	7.70E-01
		UKB	88	1.01	0.99	1.03	1.88E-01
NO_X_		Finn	80	0.94	0.79	1.13	5.30E-01
		UKB	78	1.02	1.00	1.03	1.17E-01

We also performed extensive sensitivity analyses to validate the causal association between air pollutants and the occurrence of essential hypertension (Table 2). The Cochran’s Q test in the IVW and MR Egger suggested no significant heterogeneity among these air pollution IVs in the Finn group. However, there was substantial evidence of heterogeneity in most IVs in the UKB group, which may be caused by a population overlap between hypertension and air pollution in the UKB. The primary method we used, random-effect IVW, could be fitted to the presence of heterogeneity. No apparent horizontal pleiotropy was observed using MR-Egger, as the intercept did not significantly deviate from zero, which suggested balanced pleiotropy in the univariable MR analysis.

**Table 2:** Sensitivity analyses.

Exposure	Outcome	Source	Pleiotropy			Heterogeneity			
			Egger_intercept	SE	pval	Method	Q	Q_df	Q_pval
PM2.5	Essential hypertension	Finn	1.80E-03	3.21E-03	5.77E-01	MR Egger	66.64	57	1.79E-01
						Inverse variance weighted	67.01	58	1.95E-01
PM10			7.80E-03	6.51E-03	2.40E-01	MR Egger	35.43	31	2.67E-01
						Inverse variance weighted	37.08	32	2.46E-01
NO_2_			1.35E-03	2.77E-03	6.28E-01	MR Egger	101.49	88	1.54E-01
						Inverse variance weighted	101.77	89	1.68E-01
NO_X_			-1.67E-03	2.75E-03	5.46E-01	MR Egger	68.71	78	7.65E-01
						Inverse variance weighted	69.08	79	7.80E-01
PM2.5		UKB	4.23E-04	3.79E-04	2.68E-01	MR Egger	78.30	59	4.72E-02
						Inverse variance weighted	79.96	60	4.35E-02
PM10			9.30E-04	4.70E-04	5.66E-02	MR Egger	39.41	31	1.43E-01
						Inverse variance weighted	44.40	32	7.13E-02
NO_2_			4.18E-04	3.27E-04	2.05E-01	MR Egger	121.60	86	6.96E-03
						Inverse variance weighted	123.90	87	5.75E-03
NO_X_			5.20E-04	4.09E-04	2.08E-01	MR Egger	132.11	76	7.09E-05
						Inverse variance weighted	134.91	77	5.03E-05

### MVMR analyses

**Figure 3:**
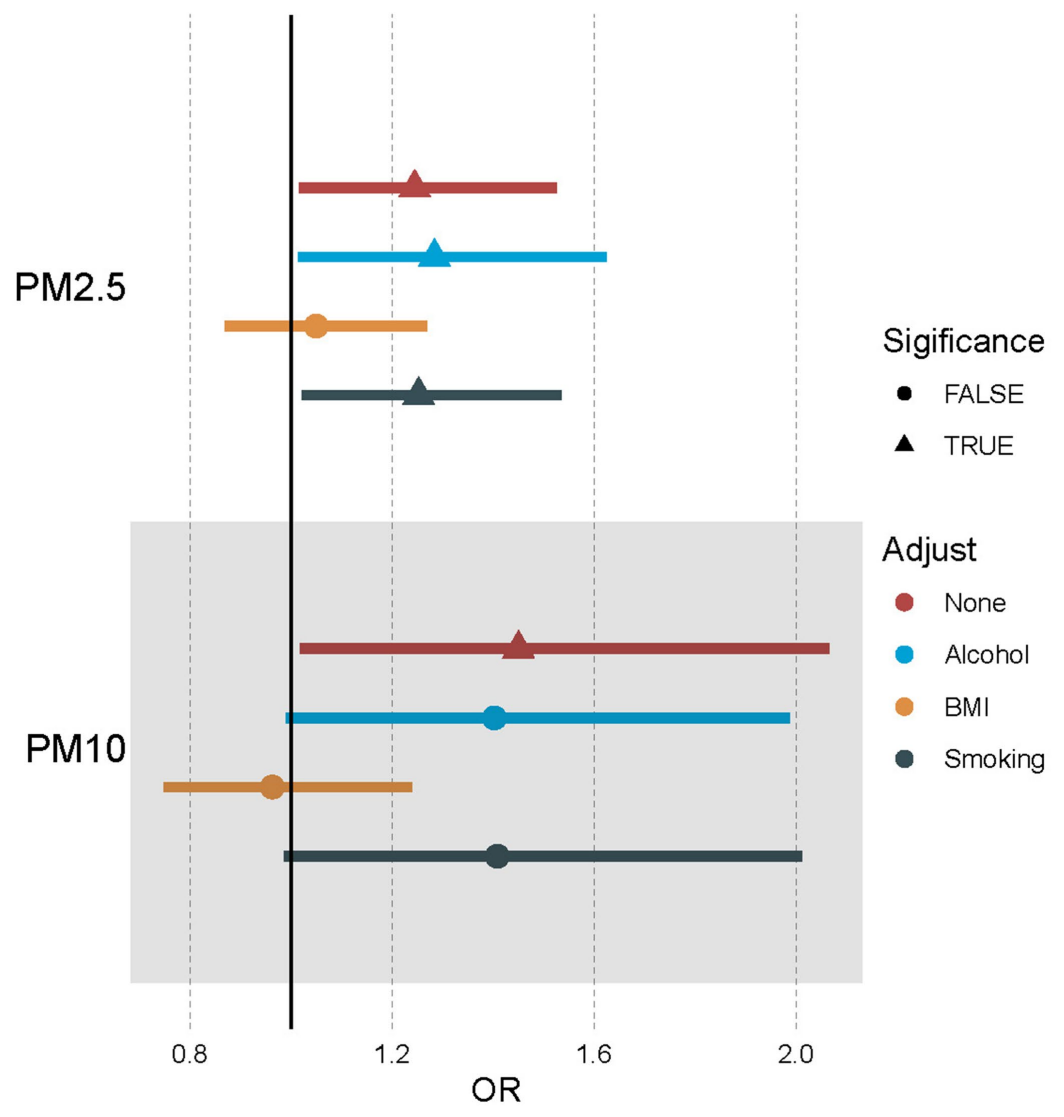
Forest plot of the association between air pollutants and hypertension using multivariable Mendelian randomization after adjusting alcohol, BMI, and smoking. OR means odds ratio. TRUE represents the causal association between the air pollutant and hypertension. FALSE represents no significant causal association between the air pollutant and hypertension.

When considering their connection to developing essential hypertension, it is crucial to understand how various factors can influence the link between exposure to pollutant particles in the atmosphere, like PM2.5 and PM10. Leveraging MVMR Analysis while incorporating confounders or mediators such as BMI, smoking, and alcohol will help us better understand these complex relations. Our results indicated that there was still a positive relationship between PM2.5 exposure and essential hypertension after adjusting for alcohol and smoking but no direct effect after adjusting for BMI (OR [95%CI] = 1.05 [0.87–1.27], *p* = 6.17E-01; Supplementary Table 7). And for PM10, no significant direct effects were detected after adjusting for alcohol, BMI, and smoking (Table 3; Figure 3; Supplementary Table 7).

**Table 3:** MVMR results for causal effects of air pollution on essential hypertension after adjusting for alcohol, BMI and smoking.

Exposure	Outcome	Adjustment	nSNP	OR	LCI	UCI	pval
PM2.5	Essential hypertension	None	59	1.24	1.02	1.53	3.46E-02
PM2.5		Alcohol	52	1.28	1.01	1.62	3.73E-02
PM2.5		BMI	40	1.05	0.87	1.27	6.17E-01
PM2.5		Smoking	58	1.25	1.02	1.54	2.99E-02
PM10		None	53	1.45	1.02	2.07	3.92E-02
PM10		Alcohol	33	1.40	0.99	1.99	5.71E-02
PM10		BMI	26	0.96	0.75	1.24	7.70E-01
PM10		Smoking	33	1.41	0.99	2.01	5.94E-02

## Discussion

Our research showed a causal relationship between increased exposure to both PM 2.5 and PM10 levels and an increase in the incidence of essential hypertension. It remained uncertain whether there was a link between essential hypertension risk and exposure to NO_2_ or NO_X_. Moreover, the increased risk of essential hypertension by PM2.5 was mediated by BMI. At the same time, BMI had the more significant mediating effect in contrast to smoking and alcohol regarding the effects of PM10 on essential hypertension.

Studies have consistently shown a positive relationship between short-term exposure to air pollution and the incidence of essential hypertension (42, 43). It was discovered through a systematic evaluation and meta-analysis report released in 2021 that there is a likelihood for individuals residing in areas where air pollution levels containing PM2.5 and PM10 for extended periods to develop hypertension, while this effect was not significant for NO_2_ and NO_X_ (15). We found similar conclusions based on our research. Moreover, we applied MVMR to correct the impacts of air pollution on hypertension by adjusting for BMI, smoking, and alcohol. BMI has the potential to mediate the relationship between hypertension and exposure to particles such as PM2.5 or PM10. Smoking and alcohol might have a mediating or synergistic effect on the relationship between PM10 and hypertension. These results suggested the complexity of the effects of air pollution and other confounding factors on hypertension.

The impact of air pollution on the risk of hypertension is a complex issue that is affected by various factors. Many previous studies have shown that BMI, smoking, and alcohol were significant risk factors for developing hypertension (28, 44). These common factors might play crucial mediating roles in air pollution and hypertension. For example, people exposed to heavy air pollution are associated with a higher risk of obesity (45). Obesity is also widely recognized as a long-established risk factor for hypertension (46), suggesting that it may likely be a significant mediating factor between air pollution and the increased risk of hypertension. Besides, obesity could also amplify the negative cardiovascular effects of PM2.5 pollution, especially concerning blood pressure and hypertension rates (47). People with obesity have increased susceptibility to the cardiovascular damage effects of air pollutants (48). For other factors, the association between smoking behavior and hypertension differed based on different levels of air pollution (49). In areas with high PM2.5, smoking was associated with a higher risk of hypertension. While in areas with low PM2.5, this was not observed, indicating that smoking might also act synergistically with air pollutants on hypertension (49).

The possible mechanisms by which air pollution increases the risk of hypertension have been widely studied (50). As the most critical air pollutant, PM has a complex mechanism related to hypertension. The most important are oxidative stress and inflammatory reactions, which are closely related and mutually induced (51). The former will promote vascular dysfunction, damage endothelial homeostasis, increase vascular permeability, and raise blood pressure (52). PM exposure has been found to result in an elevation of inflammatory cytokines and ROS levels (53), along with cellular infiltration that results in either local or systemic inflammation mediated via air-blood barrier breakdown at alveolar level (54). Additionally, hormones like cortisol, adrenaline, and noradrenaline get raised through PM, increasing the likelihood of developing hypertension (55–57). However, the mechanism by which PM2.5 and PM10 increase the risk of hypertension is currently unclear, and further research is needed to clarify it.

Our findings provided novel research insights and experimental evidence for understanding the adverse effects of air pollution on human well-being. They demonstrated the relationship between several air pollutants and primary hypertension while minimizing confounding factors and reverse causality. This could guide the screening of susceptible populations and the prevention of hypertension. Individuals with hypertension usually have a significantly higher risk of developing potentially life-threatening conditions such as heart disease and stroke (58). Consequently, we believe that our study can suggest the effect of air pollution on cardiovascular diseases to a certain degree. Additionally, conducting MVMR studies incorporating other confounding factors such as BMI, smoking, and alcohol can help inform targeted prevention strategies for cardiovascular disease in air-polluted populations.

There are some strengths to our research. Firstly, we used Mendelian randomization to establish an association between air pollution and hypertension. Secondly, we replicated the MR analysis to increase the validation. The sample size was large enough (Finn: 42,857 cases and 162,837 controls; UKB: 54,358 cases and 408,652 controls), making the estimated results as close to the actual values as possible (59).

However, some things could be improved in our study. First, we only included European ancestry, limiting the expansion of our conclusion to other races. Second, the potential bias of sample overlapping in our validated MR exists. Third, more mediators and mechanisms in which air pollutants cause hypertension must be revealed in the future. Last, genetic variants and health outcomes within UKB were associated with birth location, which could not be accounted for routine adjustments (60). This geographic structure of populations might produce biased associations for the genetic instruments.

## Data availability statement

The original contributions presented in the study are included in the article/Supplementary material, further inquiries can be directed to the corresponding authors.

## Author contributions

JW and YL wrote the manuscript. ZX and HZ supervised the study and revised the manuscript. CL, ZD, XL, NZ, and WW revised the manuscript. WW checked the language of the mansucript. All authors contributed to the article and approved the submitted version.

## Footnotes

^1^https://gwas.mrcieu.ac.uk/

^2^https://github.com/MRCIEU/TwoSampleMR

## References

[1] Daryanoosh M Goudarzi G Rashidi R Keishams F Hopke PK Mohammadi MJ. Risk of morbidity attributed to ambient PM10in the western cities of Iran. Toxin Rev. (2017) 37:313–8. doi: 10.1080/15569543.2017.1370602

[2] Wang F Wang X. Associations between PM2.5 exposure duration and physical activity intensity on the health of hypertension in urban residents of Beijing. Environ Sci Pollut Res Int. (2021) 28:29742–54. doi: 10.1007/s11356-021-12698-z, PMID: 33569688

[3] Landrigan PJ Fuller R Acosta NJR Adeyi O Arnold R Basu N(N). The lancet commission on pollution and health. Lancet. (2018) 391:462–512. doi: 10.1016/S0140-6736(17)32345-0 29056410

[4] Brook RD Rajagopalan S Pope CA III Brook JR Bhatnagar A Diez-Roux AV. Particulate matter air pollution and cardiovascular disease: an update to the scientific statement from the American Heart Association. Circulation. (2010) 121:2331–78. doi: 10.1161/CIR.0b013e3181dbece1 20458016

[5] Rajagopalan S Al-Kindi SG Brook RD. Air pollution and cardiovascular disease: JACC state-of-the-art review. J Am Coll Cardiol. (2018) 72:2054–70. doi: 10.1016/j.jacc.2018.07.099 30336830

[6] Wolf K Hoffmann B Andersen ZJ Atkinson RW Bauwelinck M Bellander T. Long-term exposure to low-level ambient air pollution and incidence of stroke and coronary heart disease: a pooled analysis of six European cohorts within the ELAPSE project. Lancet Planet Heal. (2021) 5:e620–32. doi: 10.1016/S2542-5196(21)00195-9 34508683

[7] Cohen AJ Brauer M Burnett R Anderson HR Frostad J Estep K. Estimates and 25-year trends of the global burden of disease attributable to ambient air pollution: an analysis of data from the global burden of diseases study 2015. Lancet. (2017) 389:1907–18. doi: 10.1016/S0140-6736(17)30505-6, PMID: 28408086 PMC5439030

[8] Mills KT Bundy JD Kelly TN Reed JE Kearney PM Reynolds K. Global disparities of hypertension prevalence and control: a systematic analysis of population-based studies from 90 countries. Circulation. (2016) 134:441–50. doi: 10.1161/CIRCULATIONAHA.115.018912, PMID: 27502908 PMC4979614

[9] Mills KT Stefanescu A He J. The global epidemiology of hypertension. Nat Rev Nephrol. (2020) 16:223–37. doi: 10.1038/s41581-019-0244-2 32024986 PMC7998524

[10] Ma J Chen X. Advances in pathogenesis and treatment of essential hypertension. Front Cardiovasc Med. (2022) 9:1003852. doi: 10.3389/fcvm.2022.1003852 36312252 PMC9616110

[11] Newberry SJ. Sodium and potassium intake: Effects on chronic disease outcomes and risks. Rockville (MD): (2018). Agency for Healthcare Research and Quality (US). 30125063

[12] Alpsoy S. Exercise and hypertension. Adv Exp Med Biol. (2020) 1228:153–67. doi: 10.1007/978-981-15-1792-1_10 32342456

[13] Neter JE Stam BE Kok FJ Grobbee DE Geleijnse JM. Influence of weight reduction on blood pressure: a meta-analysis of randomized controlled trials. Hypertension. (2003) 42:878–84. doi: 10.1161/01.HYP.0000094221.86888.AE 12975389

[14] Qin P Luo X Zeng Y Zhang Y Li Y Wu Y. Long-term association of ambient air pollution and hypertension in adults and in children: a systematic review and meta-analysis. Sci Total Environ. (2021) 796:148620. doi: 10.1016/j.scitotenv.2021.148620, PMID: 34274662

[15] Fuks KB Weinmayr G Foraster M Dratva J Hampel R Houthuijs D. Arterial blood pressure and long-term exposure to traffic-related air pollution: an analysis in the European study of cohorts for air pollution effects (ESCAPE). Environ Health Perspect. (2014) 122:896–905. doi: 10.1289/ehp.1307725 24835507 PMC4154218

[16] Braziene A Tamsiunas A Luksiene D Radisauskas R Andrusaityte S Dedele A. Association between the living environment and the risk of arterial hypertension and other components of metabolic syndrome. J Public Health. (2020) 42:E142–9. doi: 10.1093/pubmed/fdz046, PMID: 31234209

[17] Huang KY Yang X Liang F Liu F Li J Xiao Q. Long-term exposure to fine particulate matter and hypertension incidence in China: the China-PAR cohort study. Hypertension. (2019) 73:1195–201. doi: 10.1161/HYPERTENSIONAHA.119.12666, PMID: 31067193 PMC6656583

[18] Bowden J Holmes MV. Meta-analysis and Mendelian randomization: a review. Res Synth Methods. (2019) 10:486–96. doi: 10.1002/jrsm.1346, PMID: 30861319 PMC6973275

[19] Choi KW Chen CY Stein MB Klimentidis YC Wang MJ Koenen KC. Assessment of bidirectional relationships between physical activity and depression among adults a 2-sample Mendelian randomization study. JAMA Psychiatry. (2019) 76:399–408. doi: 10.1001/jamapsychiatry.2018.4175, PMID: 30673066 PMC6450288

[20] Sanderson E Glymour MM Holmes MV Kang H Morrison J Munafò MR. Mendelian randomization. Nature Reviews Methods Primers. (2022) 2:6. doi: 10.1038/s43586-021-00092-5, PMID: 37325194 PMC7614635

[21] Li GH Cheung CL Chung AKK Cheung BMY Wong ICK Fok MLY. Evaluation of bi-directional causal association between depression and cardiovascular diseases: a Mendelian randomization study. Psychol Med. (2022) 52:1765–76. doi: 10.1017/S0033291720003566, PMID: 33032663

[22] Burgess S Dudbridge F Thompson SG. Re: "multivariable Mendelian randomization: the use of pleiotropic genetic variants to estimate causal effects". Am J Epidemiol. (2015) 181:290–1. doi: 10.1093/aje/kwv017 25660081

[23] Burgess S Davies NM Thompson SG. Bias due to participant overlap in two-sample Mendelian randomization. Genet Epidemiol. (2016) 40:597–608. doi: 10.1002/gepi.21998, PMID: 27625185 PMC5082560

[24] Hemani G Zheng J Elsworth B Wade KH Haberland V Baird D. The MR-base platform supports systematic causal inference across the human phenome. Elife. (2018) 7:7. doi: 10.7554/eLife.34408 PMC5976434 29846171

[25] Bycroft C Freeman C Petkova D Band G Elliott LT Sharp K. The UK biobank resource with deep phenotyping and genomic data. Nature. (2018) 562:203–9. doi: 10.1038/s41586-018-0579-z, PMID: 30305743 PMC6786975

[26] Eeftens M Beelen R de Hoogh K Bellander T Cesaroni G Cirach M. Development of land use regression models for PM(2.5), PM(2.5) absorbance, PM(10) and PM(coarse) in 20 European study areas; results of the ESCAPE project. Environ Sci Technol. (2012) 46:11195–205. doi: 10.1021/es301948k 22963366

[27] Beelen R Hoek G Vienneau D Eeftens M Dimakopoulou K Pedeli X. Development of NO2 and NOx land use regression models for estimating air pollution exposure in 36 study areas in Europe – the ESCAPE project. Atmos Environ. (2013) 72:10–23. doi: 10.1016/j.atmosenv.2013.02.037

[28] van Oort S Beulens JWJ van Ballegooijen AJ Grobbee DE Larsson SC. Association of Cardiovascular Risk Factors and Lifestyle Behaviors with Hypertension: a Mendelian randomization study. Hypertension (Dallas, Tex: 1979). (2020) 76:1971–9. doi: 10.1161/HYPERTENSIONAHA.120.15761 33131310

[29] Kurki MI Karjalainen J Palta P Sipilä TP Kristiansson K Donner KM. FinnGen provides genetic insights from a well-phenotyped isolated population. Nature. (2023) 613:508–18. doi: 10.1038/s41586-022-05473-8 36653562 PMC9849126

[30] Kwok MK Kawachi I Rehkopf D Schooling CM. The role of cortisol in ischemic heart disease, ischemic stroke, type 2 diabetes, and cardiovascular disease risk factors: a bi-directional Mendelian randomization study. BMC Med. (2020) 18:363. doi: 10.1186/s12916-020-01831-3, PMID: 33243239 PMC7694946

[31] Burgess S Thompson SG. Avoiding bias from weak instruments in Mendelian randomization studies. Int J Epidemiol. (2011) 40:755–64. doi: 10.1093/ije/dyr036, PMID: 21414999

[32] Hemani G Bowden J Davey Smith G. Evaluating the potential role of pleiotropy in Mendelian randomization studies. Hum Mol Genet. (2018) 27:R195–208. doi: 10.1093/hmg/ddy163, PMID: 29771313 PMC6061876

[33] Bowden J Davey Smith G Haycock PC Burgess S. Consistent estimation in Mendelian randomization with some invalid instruments using a weighted median estimator. Genet Epidemiol. (2016) 40:304–14. doi: 10.1002/gepi.21965, PMID: 27061298 PMC4849733

[34] Verbanck M Chen CY Neale B do R. Detection of widespread horizontal pleiotropy in causal relationships inferred from Mendelian randomization between complex traits and diseases. Nat Genet. (2018) 50:693–8. doi: 10.1038/s41588-018-0099-7, PMID: 29686387 PMC6083837

[35] Burgess S Thompson SG. Interpreting findings from Mendelian randomization using the MR-egger method. Eur J Epidemiol. (2017) 32:377–89. doi: 10.1007/s10654-017-0255-x, PMID: 28527048 PMC5506233

[36] Sanderson E Davey Smith G Windmeijer F Bowden J. An examination of multivariable Mendelian randomization in the single-sample and two-sample summary data settings. Int J Epidemiol. (2019) 48:713–27. doi: 10.1093/ije/dyy262, PMID: 30535378 PMC6734942

[37] Carter AR Sanderson E Hammerton G Richmond RC Davey Smith G Heron J. Mendelian randomisation for mediation analysis: current methods and challenges for implementation. Eur J Epidemiol. (2021) 36:465–78. doi: 10.1007/s10654-021-00757-1 33961203 PMC8159796

[38] Park S Lee S Kim Y Cho S Kim K Kim YC. Causal effects of atrial fibrillation on brain white and gray matter volume: a Mendelian randomization study. BMC Med. (2021) 19:274. doi: 10.1186/s12916-021-02152-9, PMID: 34814924 PMC8611907

[39] Burgess S Thompson DJ Rees JMB Day FR Perry JR Ong KK. Dissecting causal pathways using Mendelian randomization with summarized genetic data: application to age at menarche and risk of breast Cancer. Genetics. (2017) 207:481–7. doi: 10.1534/genetics.117.300191 28835472 PMC5629317

[40] Team, R.C. R: A language and environment for statistical computing. Vienna: R Foundation for Statistical Computing (2015).

[41] Wickham H Wickham H. ggplot2: Elegant graphics for data analysis. New York: Springer-Verlag (2016).

[42] Byrd JB Morishita M Bard RL das R Wang L Sun Z. Acute increase in blood pressure during inhalation of coarse particulate matter air pollution from an urban location. J Am Soc Hypertens. (2016) 10:133–139.e4. doi: 10.1016/j.jash.2015.11.015, PMID: 26750378

[43] Hemmingsen JG Rissler J Lykkesfeldt J Sallsten G Kristiansen J Loft S. Controlled exposure to particulate matter from urban street air is associated with decreased vasodilation and heart rate variability in overweight and older adults. Part Fibre Toxicol. (2015) 12:6. doi: 10.1186/s12989-015-0081-9 25890359 PMC4374502

[44] Wu J Li T Song X Sun W Zhang Y Liu Y. Prevalence and distribution of hypertension and related risk factors in Jilin Province, China 2015: a cross-sectional study. BMJ Open. (2018) 8:e020126. doi: 10.1136/bmjopen-2017-020126 PMC5875623 29599392

[45] Zhang Z Dong B Chen G Song Y Li S Yang Z. Ambient air pollution and obesity in school-aged children and adolescents: a multicenter study in China. Sci Total Environ. (2021) 771:144583. doi: 10.1016/j.scitotenv.2020.144583, PMID: 33524680

[46] MacMahon S Cutler J Brittain E Higgins M. Obesity and hypertension: epidemiological and clinical issues. Eur Heart J. (1987) 8:57–70. doi: 10.1093/eurheartj/8.suppl_B.57, PMID: 3301356

[47] Hou J Gu J Liu X Tu R Dong X Li R. Long-term exposure to air pollutants enhanced associations of obesity with blood pressure and hypertension. Clin Nutr. (2021) 40:1442–50. doi: 10.1016/j.clnu.2021.02.029, PMID: 33740513

[48] Weichenthal S Hoppin JA Reeves F. Obesity and the cardiovascular health effects of fine particulate air pollution. Obesity. (2014) 22:1580–9. doi: 10.1002/oby.20748, PMID: 24639433 PMC4238790

[49] Chen QH Ma X Geng Y Liao J Ma L. Association between smoking and hypertension under different PM and green space exposure: a nationwide cross-sectional study. Front Public Health. (2022) 10:10. doi: 10.3389/fpubh.2022.1026648, PMID: 36466446 PMC9712966

[50] Aryal A Harmon AC Dugas TR. Particulate matter air pollutants and cardiovascular disease: strategies for intervention. Pharmacol Ther. (2021) 223:107890. doi: 10.1016/j.pharmthera.2021.107890, PMID: 33992684 PMC8216045

[51] Kim YW West XZ Byzova TV. Inflammation and oxidative stress in angiogenesis and vascular disease. J Mol Med (Berl). (2013) 91:323–8. doi: 10.1007/s00109-013-1007-3, PMID: 23430240 PMC3656485

[52] Rao X Zhong J Brook RD Rajagopalan S. Effect of particulate matter air pollution on cardiovascular oxidative stress pathways. Antioxid Redox Signal. (2018) 28:797–818. doi: 10.1089/ars.2017.7394, PMID: 29084451 PMC5831906

[53] Lawal AO. Air particulate matter induced oxidative stress and inflammation in cardiovascular disease and atherosclerosis: the role of Nrf2 and AhR-mediated pathways. Toxicol Lett. (2017) 270:88–95. doi: 10.1016/j.toxlet.2017.01.017 28189649

[54] Farina F Sancini G Longhin E Mantecca P Camatini M Palestini P. Milan PM1 induces adverse effects on mice lungs and cardiovascular system. Biomed Res Int. (2013) 2013:1–10. doi: 10.1155/2013/583513, PMID: 23509745 PMC3591224

[55] Toledo-Corral CM Alderete TL Herting MM Habre R Peterson AK Lurmann F. Ambient air pollutants are associated with morning serum cortisol in overweight and obese Latino youth in Los Angeles. Environ Health. (2021) 20:39. doi: 10.1186/s12940-021-00713-2 33832509 PMC8034084

[56] Liu L Urch B Szyszkowicz M Evans G Speck M van Huang A. Metals and oxidative potential in urban particulate matter influence systemic inflammatory and neural biomarkers: a controlled exposure study. Environ Int. (2018) 121:1331–40. doi: 10.1016/j.envint.2018.10.055, PMID: 30420132 PMC6396878

[57] Li HC Cai J Chen R Zhao Z Ying Z Wang L. Particulate matter exposure and stress hormone levels a randomized, double-blind, crossover trial of air purification. Circulation. (2017) 136:618–27. doi: 10.1161/CIRCULATIONAHA.116.026796 28808144

[58] Chobanian AV Bakris GL Black HR Cushman WC Green LA Izzo JL Jr. Seventh report of the joint National Committee on prevention, detection, evaluation, and treatment of high blood pressure. Hypertension. (2003) 42:1206–52. doi: 10.1161/01.HYP.0000107251.49515.c2 14656957

[59] Didelez V Meng S Sheehan NA. Assumptions of IV methods for observational epidemiology. Stat Sci. (2010) 25:22–40. doi: 10.1214/09-STS316

[60] Haworth S Mitchell R Corbin L Wade KH Dudding T Budu-Aggrey A. Apparent latent structure within the UK biobank sample has implications for epidemiological analysis. Nat Commun. (2019) 10:333. doi: 10.1038/s41467-018-08219-1 30659178 PMC6338768

